# Impact of Diet on Gut Microbiota in Diverticular Disease of the Colon: An Exploratory Retrospective Study

**DOI:** 10.3390/microorganisms13112428

**Published:** 2025-10-23

**Authors:** Antonio Tursi, Giorgia Procaccianti, Federica D’Amico, Rudi De Bastiani, Leonardo Allegretta, Natale Antonino, Elisabetta Baldi, Carlo Casamassima, Giovanni Casella, Mario Ciuffi, Marco De Bastiani, Lorenzo Lazzarotto, Claudio Licci, Maurizio Mancuso, Antonio Penna, Giuseppe Pranzo, Guido Sanna, Cesare Tosetti, Maria Zamparella, Marcello Picchio, Silvia Turroni

**Affiliations:** 1 Territorial Gastroenterology Service, Barletta-Andria-Trani Local Health Agency, 76123 Andria, Italy; 2 Department of Medical and Surgical Sciences, School of Medicine, Catholic University, 00168 Rome, Italy; 3 Unit of Microbiome Science and Biotechnology, Department of Pharmacy and Biotechnology, University of Bologna, 40126 Bologna, Italy; giorgia.procaccianti@unibo.it (G.P.); federica.damico8@unibo.it (F.D.); silvia.turroni@unibo.it (S.T.); 4 GIGA-CP Italian Association for Primary Care Gastroenterology, 32032 Feltre, Italy; rudeba@libero.it (R.D.B.); eli.baldi@gmail.com (E.B.); caselgio@tiscali.it (G.C.); m.ciuffi@tiscali.it (M.C.); marcomed8888@gmail.com (M.D.B.); lorenzo.lazzarotto@icloud.com (L.L.); mancuso@studiomedico11.it (M.M.);; 5 Division of Gastroenterology, “Santa Caterina Novella” Hospital, 73013 Galatina, Italy; leonardo.allegretta@tin.it; 6 Independent Researcher, 76011 Bisceglie, Italy; antoninonatale@inwind.it; 7 Independent Researcher, 76017 San Ferdinando di Puglia, Italy; carlocasamassima@hotmail.it; 8 Independent Researcher, 70043 Monopoli, Italy; licciclaudio@tin.it; 9 Independent Researcher, 70122 Bari, Italy; antoniopenna1@libero.it; 10 Ambulatory for IBD Treatment, “Valle D’Itria” Hospital, 74015 Martina Franca, Italy; giuseppepranzo63@gmail.com; 11 Division of Surgery, “P. Colombo” Hospital, Velletri, 00049 Rome, Italy; 12 IRCCS Azienda Ospedaliero-Universitaria di Bologna, 40138 Bologna, Italy

**Keywords:** diet, symptomatic uncomplicated diverticular disease (SUDD), gut microbiota, Diverticular Inflammation and Complication Assessment (DICA) classification

## Abstract

Symptomatic uncomplicated diverticular disease (SUDD) is the primary clinical manifestation of diverticular disease (DD). Although gut microbiota (GM) perturbation and dietary habits are considered important factors in the development of the disease, there is currently a lack of data on the potential relationship between diet, GM profile and SUDD. An exploratory retrospective study was conducted in a SUDD cohort of 47 patients to investigate this relationship; a diverticulosis cohort of 19 patients served as the control group. Patients were stratified by their self-reported dietary habits, i.e., Mediterranean diet, predominantly plant-based diet or omnivorous diet. GM was profiled using 16S rRNA amplicon sequencing of fecal swabs. SUDD patients following a Mediterranean or predominantly plant-based diet showed higher alpha diversity and enrichment of known fibre degraders and short-chain fatty acid producers, such as members of the *Lachnospiraceae*, *Ruminococcaceae*, *Oscillospiraceae* and *Prevotellaceae* families. This suggests that their gut (and whole-body) health is less impaired. In contrast, those following an omnivorous diet showed an increased presence of pro-inflammatory taxa, including the mucus degrader *R. torques*, which suggests impaired gut barrier function and potential systemic implications. Similar associations between GM profile and dietary habits were found when considering SUDD patients with moderate abdominal pain severity (according to visual analogue scale, VAS) and those scored as DICA 1 according to the endoscopic severity of the disease. However, no such associations or trends were observed in SUDD patients scored as DICA 2, which suggests that diet may be unable to impact GM dysbiosis as SUDD severity increases. Despite the study’s limitations, primarily its retrospective design and related biases, our findings suggest that other GM modulation tools should be employed in more severe cases of SUDD to reverse dysbiosis while alleviating symptoms.

## 1. Introduction

Thanks to the large number of colonoscopies routinely performed worldwide, diverticulosis of the colon is the most commonly detected anatomical alteration [1]. The pathogenesis of diverticular disease (DD) remains poorly defined, although some clinical risk factors and etiopathogenetic characteristics seem to be more evident [1,2,3]. In particular, it is currently hypothesized that a combination of low dietary fibre intake, gut microbiota (GM) perturbation, neuromuscular dysfunction, motility alterations, and low-grade inflammation acts together in the occurrence of the disease [1,2,3]. The prevailing paradigm in diverticulosis development focuses on low dietary fibre intake, resulting in higher intracolonic pressure and development of diverticula in areas of mucosal weakness (where vasa recta cross the colonic wall) [4]. Dietary fibre, obtained through the consumption of fruits, vegetables, and cereal grains, plays a crucial role in gut and systemic health [5]. It increases faecal mass, regularises bowel movements, and acts as a prebiotic, increasing the abundance of taxa that produce short-chain fatty acids (SCFAs) and reducing the abundance of pro-inflammatory taxa [6]. However, this paradigm has become controversial. Peery et al. found that constipation and hard stools were associated with a reduced risk of diverticulosis [7]. This paradoxical finding was linked to another, namely that there was no association between dietary fibre intake and the risk of diverticulosis [7,8].

Meat consumption appears to follow a similar pattern. Aldoori et al. found a significant association between red meat intake and an increased risk of DD, despite there being no dose–response relationship [9]. However, Peery et al. found no such association [7]. On the other hand, red meat intake, particularly unprocessed red meat, has been associated with an increased risk of developing acute diverticulitis [10]. These conflicting results, regarding both fibre and meat, could be due to various study-related biases (e.g., study design, population factors, cultural dietary patterns, diagnostic methods, and endpoints).

Most of the research has focused on the relationship between diet and diverticulosis or acute diverticulitis. Conversely, little research has been conducted into the potential relationship between diet and the occurrence of symptomatic uncomplicated diverticular disease (SUDD). SUDD is the main form of DD, affecting around 20% of patients with diverticulosis, and significantly increasing the risk of acute diverticulitis (it is estimated that this risk is double that of patients with diverticulosis alone) [11]. Therefore, the impact of SUDD on national health systems could be significant. Even in the absence of well-structured data, it is estimated that the management of SUDD and the prevention of acute diverticulitis in these patients could cost up to 400 million euros [12]. These epidemiological data emphasize the need to better understand the pathogenesis of SUDD, including its relationship with risk factors. As with other functional disorders (e.g., irritable bowel syndrome and inflammatory bowel disease) [13], SUDD is characterized by GM dysbiosis, with over-representation of potentially harmful taxa (in particular, Proteobacteria, *Streptococcaceae* and *Megasphaera*) [14], which could contribute to inflammation and symptom exacerbation. Interestingly, GM changes are closely associated with the severity of abdominal pain [14,15] and the endoscopic severity of the disease [14], and can largely be reversed by treatment with sodium butyrate [16]. This treatment also resulted in an increase in SCFA producers, such as *Lachnospiraceae* and *Phascolarctobacterium*. These bacteria could contribute to improved gut and whole-body health by enhancing gut barrier function, promoting immunomodulatory responses and facilitating healthy gut–brain communication [17]. Currently, no data are available regarding the interaction between GM profile and diet in SUDD.

The aim of this exploratory retrospective study was to test the associations between GM profile (in terms of diversity and composition) and dietary habits in SUDD, also according to endoscopic disease severity. Patients with asymptomatic diverticulosis (AD) were used as the control group. As with healthy subjects [18], we expect diet to impact the GM structure of SUDD patients, but to a lesser extent as disease severity increases.

## 2. Materials and Methods

We conducted a retrospective analysis of stool samples from patients with SUDD who were being treated by general practitioners and territorial gastroenterologists in primary care. The samples were collected using faecal swabs for microbiological studies between 1 March 2022 and 1 March 2023. They were then stored at −80 °C at the Unit of Microbiome Science and Biotechnology in the Department of Pharmacy and Biotechnology at the University of Bologna in Bologna, Italy. Faecal swabs were collected using the eNAT System (Copan, Brescia, Italy), which preserves nucleic acids for up to four weeks at room temperature. The interchangeability of faecal sampling and swabbing for assessing GM structure has been demonstrated elsewhere [19,20]. Abdominal pain severity was measured using a 10-point visual analogue scale (VAS). Diverticulosis was scored according to the Diverticular Inflammation and Complication Assessment (DICA) classification [21,22]. As a control group, patients with AD were identified, whose faecal samples were collected using the same system during the same period.

The study was conducted in accordance with clinical practice guidelines and the principles of the Declaration of Helsinki. All patients gave written informed consent before undergoing endoscopy and/or computed tomography (CT) scan and/or faecal sampling. Ethic committee approval for this retrospective study was obtained from the Azienda Ospedaliero-Universitaria “Ospedali Riuniti”, Foggia, Italy (PROT. 164/CE/2023, 23 October 2023).

### 2.1. Inclusion Criteria

Inclusion criteria were: males and females aged over 18; diagnosis of SUDD, defined as left-lower and long-lasting (≥24 h) moderate-to-severe quadrant pain in patients with diverticulosis [23]; colonic DD diagnosed by colonoscopy and scored according to the DICA classification [21,22] during the 6 months prior to enrolment; possibility of retrospectively reconstructing symptoms (in particular, the severity of abdominal pain using VAS); bowel habits according to the Bristol stool form scale; type of diet followed, as self-reported by patients: Mediterranean diet [24], predominantly plant-based diet (a diet excluding meat, fresh or processed, including cured meats, and fish, but including animal-derived products such as dairy, eggs and honey) [25], or omnivorous diet (including plant- and animal-based foods) [26]; and faecal microbiota assessment performed at the Unit of Microbiome Science and Biotechnology, Department of Pharmacy and Biotechnology, University of Bologna (Bologna, Italy).

### 2.2. Exclusion Criteria

Exclusion criteria were: current or previous diagnosis (by abdominal CT and/or ultrasonography) of acute diverticulitis (defined as inflammation of the colonic wall harbouring diverticula with fat stranding, with or without complications such as abscesses, stenosis or fistulas, namely uncomplicated or complicated diverticulitis) [1]; inflammatory bowel disease; ischemic colitis; prior colonic resection; patients with severe liver failure (Child-Pugh C); patients with severe kidney failure; pregnant women; women of childbearing potential not using a highly effective method of contraception; patients currently using or who have received any laxative agents <4 weeks prior to enrolment; patients currently using or who have received any mesalamine compounds <4 weeks prior to enrolment; patients currently using or who have received any probiotic agents <4 weeks prior to enrolment; use of non-steroidal anti-inflammatory drugs (except for acetyl-salicylic acid ≤ 100 mg/day) <4 weeks prior to enrolment; patients treated with antibiotics (including those not absorbed) <4 weeks prior to enrolment; patients with a history of cancer, of any origin, at the time of stool collection, and/or under treatment with chemotherapy and/or radiotherapy; a history of alcohol, drug, or chemical abuse; and patients with a current or recent (≤3 months) episode of COVID-19 [27] at the time of the stool collection.

### 2.3. Endpoints

The primary endpoint was to profile the GM in patients with SUDD and AD according to their self-reported dietary habits (Mediterranean diet, predominantly plant-based diet, or omnivorous diet). The secondary endpoint was to investigate correlations among GM, dietary patterns, and other patient metadata in SUDD, namely abdominal pain severity (the main symptom of SUDD) as measured by VAS, and the endoscopic severity of the disease as measured by DICA.

### 2.4. Microbial DNA Extraction and 16S rRNA Amplicon Sequencing

Microbial DNA was extracted from the faecal swabs as previously described [14]. Briefly, the swabs were vortexed and centrifuged at 13,000 rpm for 10 min at 4 °C. The resulting pellets were resuspended in 1 mL of lysis buffer (500 mM NaCl, 50 mM Tris-HCl pH 8.0, 50 mM EDTA, and 4% SDS) and subjected to three rounds of bead-beating in a FastPrep instrument (MP Biomedicals, Irvine, CA, USA) at 5.5 movements/s for 1 min, in the presence of four 3-mm glass beads and 0.5 g of 0.1-mm zirconia beads (BioSpec Products, Bartlesville, OK, USA). After incubation at 95 °C for 15 min, the samples were centrifuged at 13,000 rpm for 5 min. Then, 260 μL of 10 M ammonium acetate was added to the supernatant, followed by incubation on ice for 5 min and centrifugation at 13,000 rpm for 10 min. Each sample was then incubated with one volume of isopropanol and left to stand on ice for 30 min. The nucleic acid precipitate was washed with 70% ethanol, resuspended in 100 μL of TE buffer (10 mM Tris-HCl, 1 mM EDTA, pH 8.0) and treated with 2 μL of 10 mg/mL DNase-free RNase at 37 °C for 15 min. DNA was then purified using the DNeasy Blood and Tissue Kit (QIAGEN, Hilden, Germany) following the manufacturer’s protocol. DNA concentration and quality were assessed using a NanoDrop ND-1000 spectrophotometer (NanoDrop Technologies, Wilmington, DE, USA).

For library preparation, the V3–V4 hypervariable regions of the 16S rRNA gene were amplified using 341F and 785R primers containing Illumina adapter overhang sequences [19]. Fragment amplification was performed using KAPA HiFi HotStart ReadyMix (Roche, Basel, Switzerland), with the following thermal cycle: 3 min at 95 °C, 25 cycles of 30 s at 95 °C, 30 s at 55 °C, and 30 s at 72 °C, and a final step of 5 min at 72 °C. After purification using a magnetic bead-based clean-up system (Agencourt AMPure XP, Beckman Coulter, Brea, CA, USA), limited-cycle PCR was performed using Nextera technology to obtain the indexed library, which was then purified again. Final libraries were prepared by pooling all samples at an equimolar concentration of 4 nM. Denaturation and dilution to 5 pM were performed prior to sequencing on an Illumina MiSeq platform using a 2 × 250 bp paired-end protocol according to the manufacturer’s instructions (Illumina, San Diego, CA, USA).

### 2.5. Bioinformatics and Statistical Analysis

The raw sequences were processed using a pipeline that combined PANDASeq [28] and QIIME 2 [29]. After filtering for length and quality, the reads were clustered into amplicon sequence variants (ASVs) using DADA2 [30]. Taxonomic assignment was performed using the VSEARCH algorithm [31] against the SILVA database (August 2020 release) [32]. Alpha diversity was assessed using various metrics, such as the Shannon index, the number of observed ASVs, and Faith’s phylogenetic diversity. Bera diversity was assessed using weighted and unweighted UniFrac distances, which were then used for Principal Coordinates Analysis (PCoA) plots.

All statistical analyses were performed using R software (version 4.2.3). Group differences in demographic, anthropometric and clinical characteristics were assessed using a Kruskal–Wallis test or a Mann–Whitney test for ordinal variables and Fisher’s exact test for categorical variables. For GM, PCoA plots were generated using the “vegan” (https://cran.r-project.org/package=vegan, last accessed on 18 October 2025) and “Made4” [33] packages. Data separation was tested using a permutation test with pseudo-F ratio (function “Adonis” in “vegan”). The contribution of covariates to the ordination space was determined using the function ‘envfit’ of vegan. Group differences in alpha diversity and relative taxon abundance were assessed using a Kruskal–Wallis test, followed by post hoc Wilcoxon tests. *p*-values were adjusted using the Benjamini–Hochberg method. A false discovery rate (FDR) ≤ 0.05 was considered statistically significant, and FDR ≤ 0.1 was considered to indicate a trend.

## 3. Results

### 3.1. Gut Microbiota Profiling in Patients with Diverticular Disease and Different Dietary Habits

According to the inclusion and exclusion criteria, 47 SUDD patients (the study group) and 19 AD patients (the control group) were identified. The demographic, anthropometric, and clinical characteristics of the enrolled patients are reported in Supplementary Table S1. The two groups did not differ in any of the characteristics assessed (of course, abdominal pain was only recorded in the SUDD group by VAS). A mean of 12,698 (±5734 SD) high-quality 16S rRNA gene sequences were obtained per patient.

**Figure 1:**
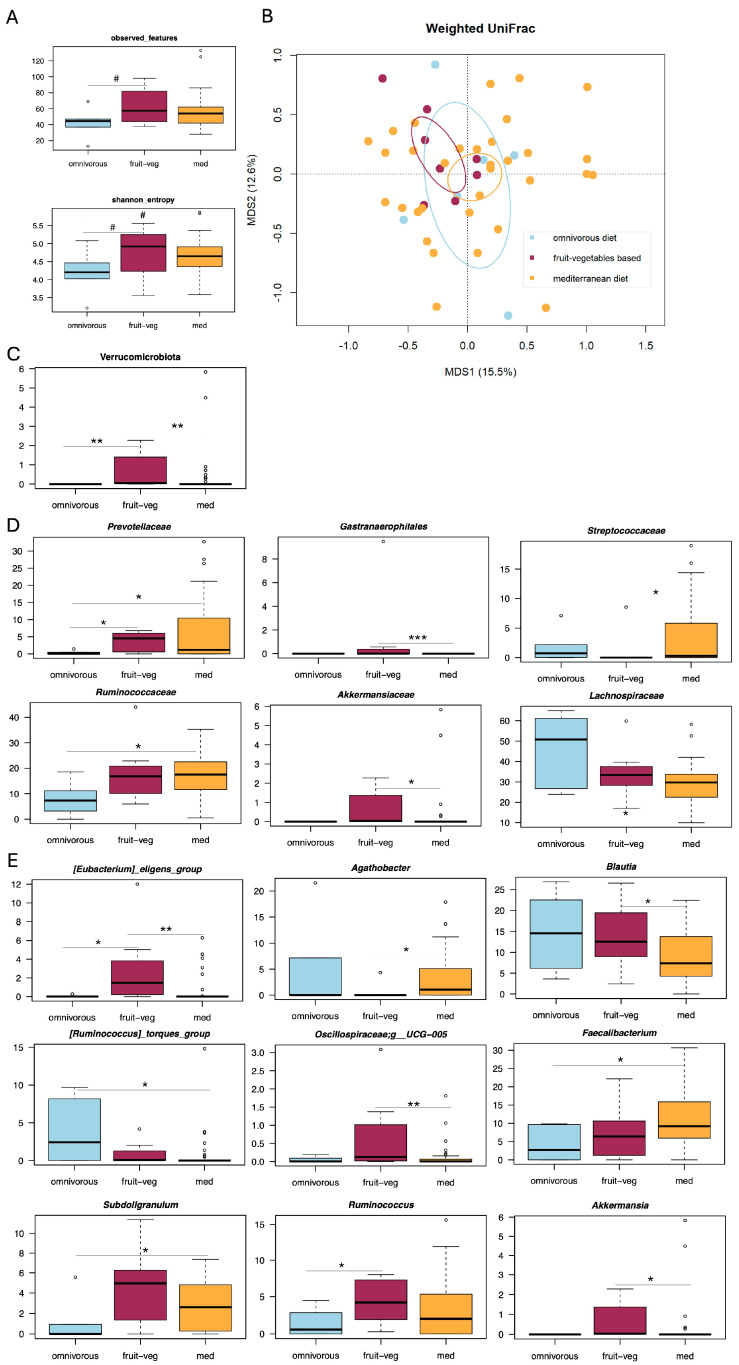
Gut microbiota profile of SUDD patients with different dietary habits. (**A**) Boxplots showing the distribution of alpha diversity, as estimated by the number of observed features and Shannon entropy, in the gut microbiota of SUDD patients who followed a Mediterranean diet (med), a predominantly plant-based diet (fruit-veg) or an omnivorous diet (omnivorous). Wilcoxon test, # *p* ≤ 0.1. (**B**) Principal Coordinates Analysis (PCoA) based on weighted UniFrac distances between the study groups. Ellipses include 95% confidence area based on the standard error of the weighted average of sample coordinates. No significant separation was found (Adonis test, *p* = 0.229). Boxplots showing the relative abundance distribution of phyla (**C**), families (**D**), and genera (**E**) differentially represented between groups. Wilcoxon test, * *p* ≤ 0.05; ** *p* ≤ 0.01; *** *p* ≤ 0.001.

Of 47 SUDD patients, 33 (70.2%) followed a Mediterranean diet, 8 (17.0%) followed a predominantly plant-based diet, and 6 (12.8%) followed an omnivorous diet (see Supplementary Table S2 for the demographic, anthropometric and clinical characteristics of the three dietary groups). In this cohort, there was a tendency towards higher alpha diversity in patients following a Mediterranean or predominantly plant-based diet (Wilcoxon test, *p* ≤ 0.13) (Figure 1A). However, no separation between groups was found in the weighted and unweighted UniFrac-based PCoA (Adonis test, *p* = 0.23) (Figure 1B and Supplementary Figure S1). Compositionally (Figure 1C–E), SUDD patients who followed a Mediterranean diet were enriched in the families *Prevotellaceae*, *Streptococcaceae*, and *Ruminococcaceae*, and the genera *Agathobacter*, *Faecalibacterium*, and *Subdoligranulum* (Wilcoxon test, *p* ≤ 0.05). Patients following a predominantly plant-based diet were enriched in the phylum Verrucomicrobiota, the families *Gastranaerophilales*, *Prevotellaceae*, and *Akkermansiaceae*, and the genera *[Eubacterium] eligenes* group, *Blautia*, *Oscillospiraceae UCG-005* and *Ruminococcus* (*p* ≤ 0.05). Finally, SUDD patients who followed an omnivorous diet were enriched in the *Lachnospiraceae* family and its genus *[Ruminococcus] torques* group (*p* ≤ 0.05). All analyses were repeated excluding SUDD patients who experienced disease recurrence within 6 months of diagnosis (unpublished data). The main findings were confirmed (see Supplementary Figure S2).

**Figure 2:**
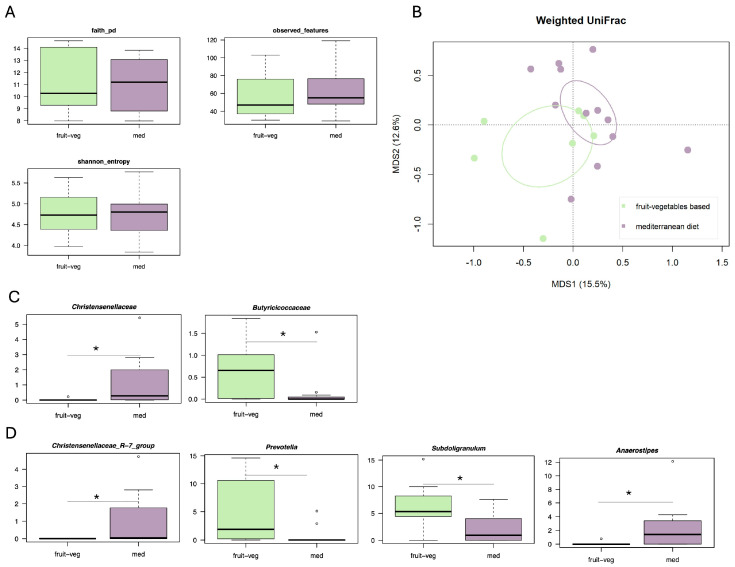
Gut microbiota profile of AD patients with different dietary habits. (**A**) Boxplots showing the distribution of alpha diversity, as estimated by Faith’s phylogenetic diversity (faith_pd), the number of observed features and Shannon entropy, in the gut microbiota of AD patients who followed either a Mediterranean diet (med) or a predominantly plant-based diet (fruit-veg). No significant differences were found (Wilcoxon test, *p* > 0.05). (**B**) Principal Coordinates Analysis (PCoA) based on weighted UniFrac distances between the study groups. Ellipses include 95% confidence area based on the standard error of the weighted average of sample coordinates. A trend towards separation was found (Adonis test, *p* = 0.07). Boxplots showing the relative abundance distribution of families (**C**) and genera (**D**) differentially represented between groups. Wilcoxon test, * *p* ≤ 0.05.

Regarding the control group, of the 19 AD patients, 12 (63.2%) followed a Mediterranean diet, and 7 (36.8%) followed a predominantly plant-based diet (see Supplementary Table S3 for the demographic, anthropometric and clinical characteristics of the three dietary groups). In this cohort, no differences were observed in alpha diversity (Figure 2A). A trend towards separation was seen in the weighted UniFrac-based PCoA (Adonis test, *p* = 0.07) (Figure 2B), but not in the unweighted UniFrac-based PCoA (*p* = 0.192) (Supplementary Figure S1). Compositionally (Figure 2C,D), AD patients who followed a Mediterranean diet were enriched in the *Christensenellaceae* family, and the genera *Christensenellaceae R-7* group and *Anaerostipes* (Wilcoxon test, *p* ≤ 0.05). Patients following a predominantly plant-based diet were enriched in the *Butyricicoccaceae* family, and the genera *Prevotella* and *Subdoligranulum* (*p* ≤ 0.05).

### 3.2. Correlations Between Gut Microbiota, Dietary Patterns and Abdominal Pain Severity in SUDD

**Figure 3:**
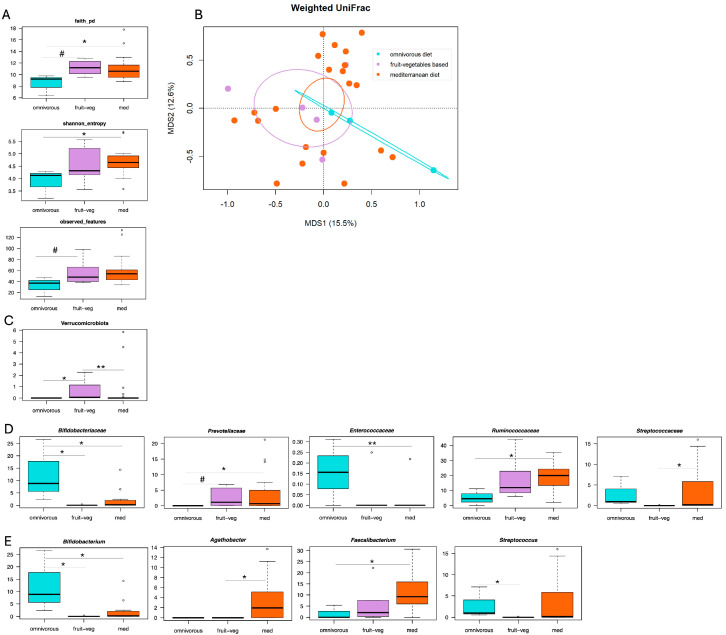
Correlations between gut microbiota, dietary patterns and abdominal pain severity in SUDD. (**A**) Boxplots showing the distribution of alpha diversity, as estimated by Faith’s phylogenetic diversity (faith_pd), the number of observed features and Shannon entropy, in the gut microbiota of patients with moderate SUDD (visual analog scale scores 4–7) who followed a Mediterranean diet (med), a predominantly plant-based diet (fruit-veg) or an omnivorous diet (omnivorous). Wilcoxon test, # *p* ≤ 0.1; * *p* ≤ 0.05. (**B**) Principal Coordinates Analysis (PCoA) based on weighted UniFrac distances between the study groups. Ellipses include 95% confidence area based on the standard error of the weighted average of sample coordinates. A trend towards separation was found (Adonis test, *p* = 0.13). Boxplots showing the relative abundance distribution of phyla (**C**), families (**D**), and genera (**E**) differentially represented between groups. Wilcoxon test, # *p* ≤ 0.1; * *p* ≤ 0.05; ** *p* ≤ 0.01.

SUDD patients were further stratified by abdominal pain severity (as estimated by VAS), and the above analyses were repeated. Due to the limited sample size, only patients with moderate SUDD (VAS scores 4–7) were considered. Of these patients, 21 (72.4%) followed a Mediterranean diet, 5 (17.2%) followed a predominantly plant-based diet, and 3 (10.4%) followed an omnivorous diet. Patients who followed a Mediterranean or predominantly plant-based diet showed (or tended to show) higher alpha diversity than the omnivorous group (Wilcoxon test, *p* ≤ 0.13) (Figure 3A). Weighted and unweighted UniFrac-based PCoA revealed a tendency for separation between the groups (Adonis test, *p* ≤ 0.13) (Figure 3B and Supplementary Figure S1). Compositionally (Figure 3C–E), patients who followed a Mediterranean diet were enriched in the families *Prevotellaceae*, *Ruminococcaceae*, and *Streptococcaceae*, and the genera *Agathobacter* and *Faecalibacterium* (Wilcoxon test, *p* ≤ 0.05). Those who followed a predominantly plant-based diet were enriched in the phylum Verrucomicrobiota and tended to be enriched in the *Prevotellaceae* family (*p* ≤ 0.08). Finally, patients following an omnivorous diet were enriched in the families *Bifidobacteriaceae* and *Enterococcaceae*, and the genera *Bifidobacterium* and *Streptococcus* (*p* ≤ 0.05).

### 3.3. Correlations Between Gut Microbiota, Dietary Patterns and DICA Classification in SUDD

Finally, SUDD patients were stratified according to DICA severity, and the above analyses were repeated. Due to the limited sample size, only patients scored as DICA 1 or DICA 2 were considered. In the DICA 1 group, 23 patients (62.2%) followed a Mediterranean diet, 8 (21.6%) followed a predominantly plant-based diet, and 6 (16.2%) followed an omnivorous diet. In the DICA2 group, 8 patients (61.5%) followed a Mediterranean diet, and 5 (38.5%) followed a predominantly plant-based diet.

**Figure 4:**
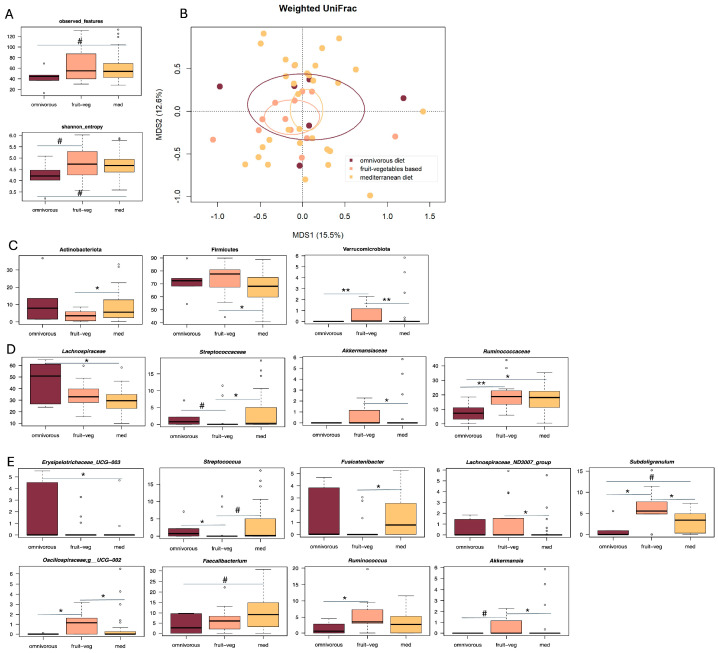
Correlations between gut microbiota, dietary patterns and DICA classification in SUDD. (**A**) Boxplots showing the distribution of alpha diversity, as estimated by the number of observed features and Shannon entropy, in the gut microbiota of SUDD patients scored as DICA1 who followed a Mediterranean diet (med), a predominantly plant-based diet (fruit-veg) or an omnivorous diet (omnivorous). Wilcoxon test, # *p* ≤ 0.1. (**B**) Principal Coordinates Analysis (PCoA) based on weighted UniFrac distances between the study groups. Ellipses include 95% confidence area based on the standard error of the weighted average of sample coordinates. A trend towards separation was found (Adonis test, *p* = 0.08). Boxplots showing the relative abundance distribution of phyla (**C**), families (**D**), and genera (**E**) differentially represented between groups. Wilcoxon test, # *p* ≤ 0.1; * *p* ≤ 0.05; ** *p* ≤ 0.01.

In the DICA 1 group, patients who followed a Mediterranean or predominantly plant-based diet tended to show higher alpha diversity than those in the omnivorous group (Wilcoxon test, *p* ≤ 0.16) (Figure 4A). Weighted UniFrac-based PCoA revealed a tendency for separation between the groups (Adonis test, *p* = 0.08) (Figure 4B). No trends were found in the unweighted UniFrac-based PCoA (*p* = 0.38) (Supplementary Figure S1). Compositionally (Figure 4C–E), patients who followed a Mediterranean diet were or tended to be enriched in the phylum Actinobacteriota, the family *Streptococcaceae* and the genera *Streptococcus* and *Fusicatenibacter* compared to those following a predominantly plant-based diet, and in the family *Ruminococcaceae* and the genera *Subdoligranulum* and *Faecalibacterium* compared to those following an omnivorous diet (Wilcoxon test, *p* ≤ 0.07). Those who followed a predominantly plant-based diet were enriched in the phyla Firmicutes and Verrucomicrobiota, the families *Akkermansiaceae* and *Ruminococcaceae*, and the genera *Lachnospiraceae ND3007* group, *Subdoligranulum*, *Oscillospiraceae UCG-002*, *Ruminococcus* and *Akkermansia* compared to the other groups (*p* ≤ 0.06). Finally, patients following an omnivorous diet were enriched in the family *Lachnospiraceae* and the genus *Erysipelotrichaceae UCG-003* compared to those following a Mediterranean diet, and in the family *Streptococcaceae* and the genus *Streptococcus* compared to those following a predominantly plant-based diet (*p* ≤ 0.06).

No significant differences or trends were observed in alpha or beta diversity in the DICA 2 group (Adonis test, *p* ≥ 0.46) (Supplementary Figure S3).

## 4. Discussion

The impact of diet on the GM, as well as its clinical significance, is the subject of active debate today [34]. For instance, there appears to be an intriguing correlation between diet, GM profile and the severity of symptoms in conditions such as irritable bowel syndrome and inflammatory bowel disease [35,36]. This could inform potential dietary interventions aimed at restoring GM dysbiosis and improving health. However, no data are currently available for SUDD.

Here, we demonstrated that GM perturbations in SUDD could be associated with dietary habits. As expected, adopting a healthier dietary pattern (i.e., a Mediterranean or predominantly plant-based diet) resulted in healthy-like signatures, namely higher alpha diversity and greater proportions of beneficial taxa. The latter included well-known fibre degraders and SCFA producers, such as members of the *Lachnospiraceae*, *Ruminococcaceae*, *Oscillospiraceae* and *Prevotellaceae* families. These taxa typically dominate the GM profile of a healthy adult and provide ecosystem services that are instrumental to health, primarily mediated by SCFAs. These services include improving gut barrier integrity, having immuno-modulatory and anti-inflammatory effects, and improving the health of the gut–brain axis [17,37,38]. These services could be particularly beneficial in the context of SUDD, contributing to the control of symptom severity. Conversely, an omnivorous diet was associated with an increased presence of the pro-inflammatory, mucus-degrading bacterium *R. torques*. This microorganism has previously been suggested as a potential irritable bowel syndrome biomarker and has been associated with increased symptom severity, probably also through impaired tryptamine production [39,40]. As demonstrated in other functional disorders, including inflammatory bowel disease [41], the presence of this mucus degrader could contribute to leaky gut syndrome and the development of a pro-inflammatory environment, thereby exacerbating SUDD symptoms. Notably, these associations were largely confirmed in the group of SUDD patients with moderate VAS and in those classified as DICA 1, which corroborates the impact of diet on the GM profile. Adopting a Mediterranean or predominantly plant-based diet was once again found to be associated with higher proportions of beneficial taxa (including the SCFA producers belonging to the families *Lachnospiraceae* and *Ruminococcaceae*), while an omnivorous diet resulted in higher proportions of potentially harmful bacteria, including *Enterococcaceae* and *Erysipelotrichaceae*. The latter are known to be pro-inflammatory and contribute to gut mucosal injury and immune defence dysfunction [42,43]. In contrast, no associations or trends were found in SUDD patients scored as DICA 2, suggesting that the impact of diet is likely to be less significant as endoscopic disease severity (and disease-driven dysbiosis) increases. This indicates that other strategies for modulating the GM should be employed in these patients to decrease or overcome GM resilience. Furthermore, it is hypothesized that, in DICA 2 patients, factors other than diet, such as inflammation, may influence outcomes. Indeed, it has recently been shown that mesalazine, an anti-inflammatory drug, is the only interventional approach capable of preventing the occurrence of acute diverticulitis in these patients [44].

This study has several limitations. The main limitation is its retrospective design, which meant that detailed dietary information could not be collected. Other limitations include the small sample size, particularly for certain patient groups, which sometimes led to borderline significance and an increased risk of overstatements, as well as the inability to control for potential confounders. Additionally, retrospective studies are prone to sample heterogeneity, but strict inclusion/exclusion criteria were adopted. Furthermore, bulk stool was unavailable, which would have been preferable also for conducting other analyses, such as metabolomics. However, the interchangeability of faecal sampling and swabbing for GM assessment has been demonstrated [19,20]. Finally, analyses could not be conducted on patients with severe VAS or those classified as DICA 3.

## 5. Conclusions

Our exploratory retrospective study suggests that, in the absence of major severity, the diet can still be used to steer the GM configuration towards a healthier profile (capable of producing SCFAs and maintaining gut homeostasis) that is less prone to inflammation and/or complications (including those related to mucus degradation and aberrant immune responses). Conversely, in cases of more severe disease and related dysbiosis (such as SUDD patients with a DICA 2 endoscopic score and a higher VAS score), it is reasonable to assume that other GM modulation tools should be employed, including probiotics or postbiotics such as SCFAs. In particular, butyrate supplementation [16] could be promising in controlling both symptoms and disease severity, while reducing or overcoming GM resilience.

Further prospective studies enrolling larger cohorts with adequate representation of all stratified groups, and controlling for potential confounders, are needed to validate and extend our findings. These studies should collect detailed dietary information using validated dietary assessment tools (e.g., food frequency questionnaires and/or 24-h recalls) and conduct functional analyses (e.g., metagenomics and metabolomics) and in vitro experiments to provide mechanistic insights, particularly with regard to SCFA production, mucus degradation and immune stimulation. Once this knowledge has been acquired, the next step should be to design appropriate interventional studies with the ultimate goal of improving the clinical management of SUDD.

## Data Availability

The raw sequence reads are available at the National Center for Biotechnology Information Sequence Read Archive (NCBI SRA; BioProject ID: PRJNA1216941).

## Supplementary Materials

The following supporting information can be downloaded at: https://www.mdpi.com/article/10.3390/microorganisms13112428/s1, Figure S1: Beta diversity of the study groups; Figure S2: Gut microbiota profile of non-relapsing SUDD patients with different dietary habits; Figure S3: Correlations between gut microbiota, dietary patterns and DICA classification in SUDD; Table S1: Demographic, anthropometric and clinical characteristics of SUDD and AD patients; Table S2: Demographic, anthropometric and clinical characteristics of SUDD patients stratified by dietary habit; Table S3: Demographic, anthropometric and clinical characteristics of AD patients stratified by dietary habit.

## Abbreviations

The following abbreviations are used in this manuscript:

GM	Gut Microbiota
DD	Diverticular Disease
DICA	Diverticular Inflammation and Complication Assessment
SUDD	Symptomatic Uncomplicated Diverticular Disease
