# Supplementary material for: Impact of Diet on Gut Microbiota in Diverticular Disease of the Colon: An Exploratory Retrospective Study
Source: Microorganisms. 2025 Oct 23;13(11):2428. doi: 10.3390/microorganisms13112428 (PMC12654463; doi:10.3390/microorganisms13112428)
Supplement: Supplementary file 1 [file microorganisms-13-02428-s001.zip › microorganisms-3876501-supplementary.pdf]

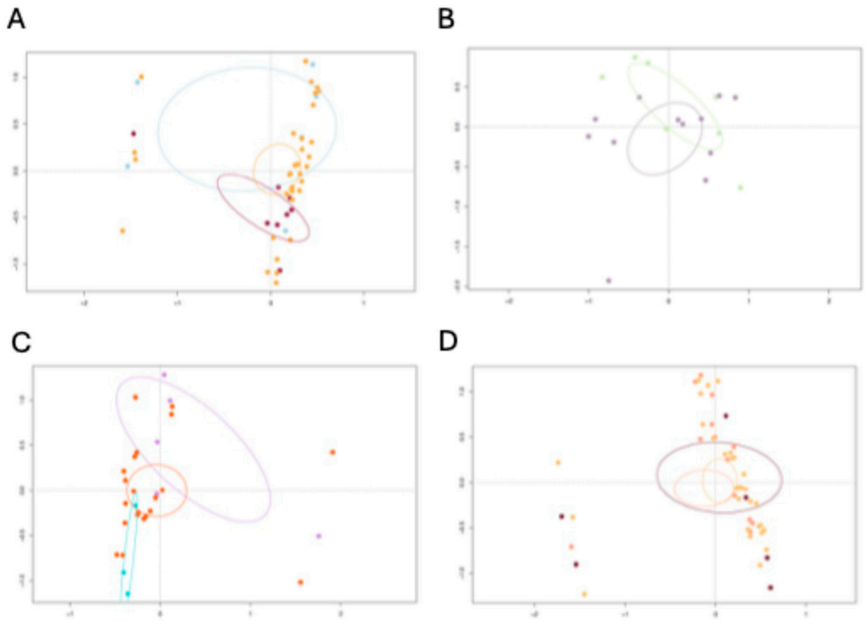

Supplementary Figure S1. Beta diversity of the study groups. Principal Coordinates Analysis (PCoA) based on unweighted UniFrac distances between the patient groups (A, SUDD patients; B, AD patients; C, SUDD patients with moderate abdominal pain severity; D, SUDD patients scored as DICA1) stratified by dietary habit (Mediterranean diet (med) or a predominantly plant-based diet (fruit-veg) or an omnivorous diet (omnivorous)). For colour legend, please refer to the main figures (Figure 1 to 4). The first and second axes are plotted. Ellipses include 95% confidence area based on the standard error of the weighted average of sample coordinates. The separation was tested using an Adonis test (A, p=0.23; B, p=0.192; C, p=0.1; D, p=0.38).

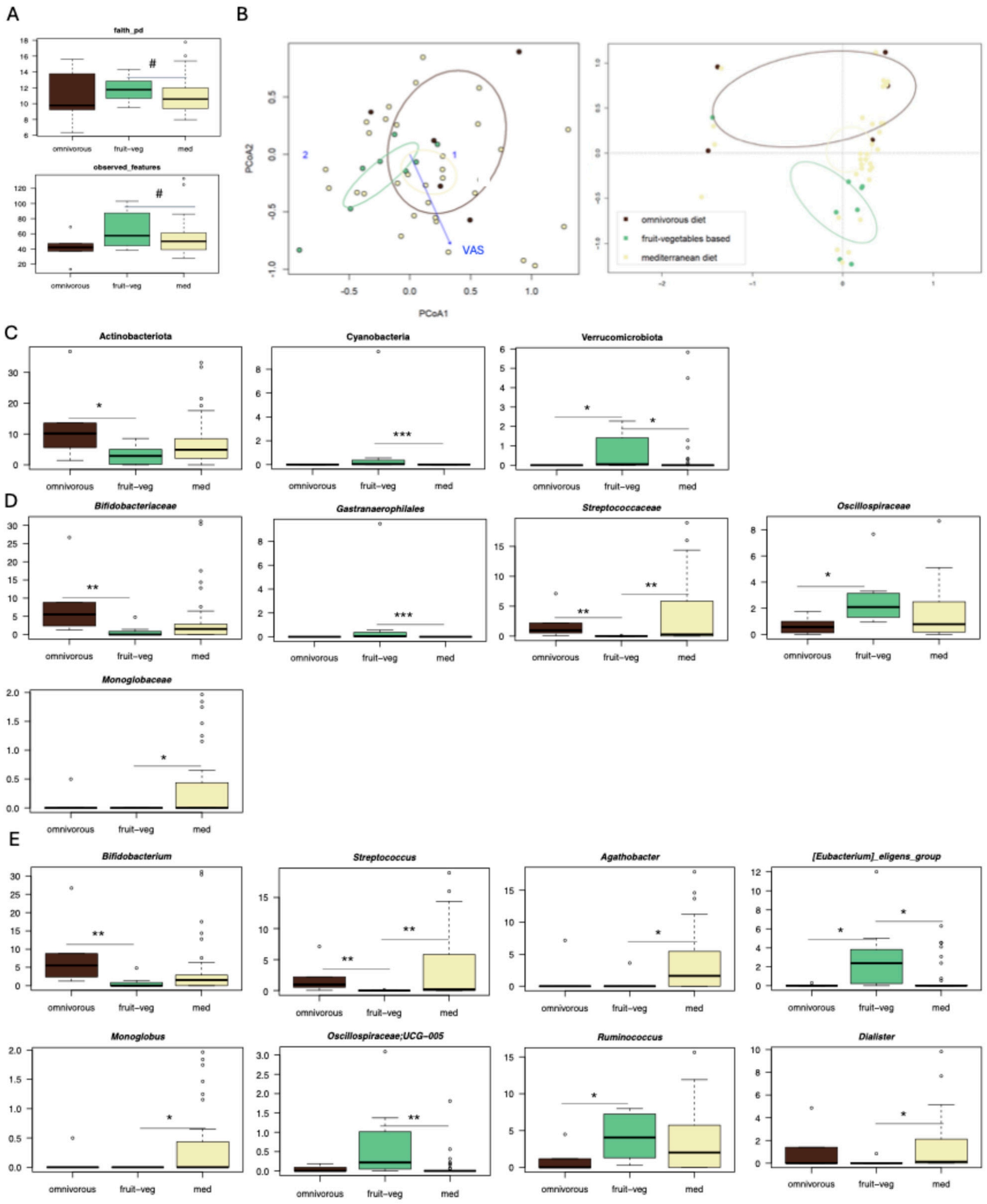

Supplementary Figure S2. Gut microbiota profile of non-relapsing SUDD patients with different dietary habits. A, Boxplots showing the distribution of alpha diversity, as estimated by Faith's phylogenetic diversity (faith_pd) and the number of observed features, in the gut microbiota of SUDD patients who did not experience SUDD recurrence within 6 months, following a Mediterranean diet (med) or a predominantly plant-based diet (fruit-veg) or an omnivorous diet (omnivorous). Wilcoxon test, #p≤0.1. B, Principal Coordinates Analysis (PCoA) based on weighted UniFrac (left) and unweighted UniFrac (right) distances between the study groups. Ellipses include 95% confidence area based on the standard error of the weighted average of sample coordinates. A trend towards separation was found (Adonis test,

p≤0.129). Boxplots showing the relative abundance distribution of phyla (C), families (D), and genera (E) differentially represented between groups. Wilcoxon test, *p≤0.05; **p≤0.01; ***p≤0.001.

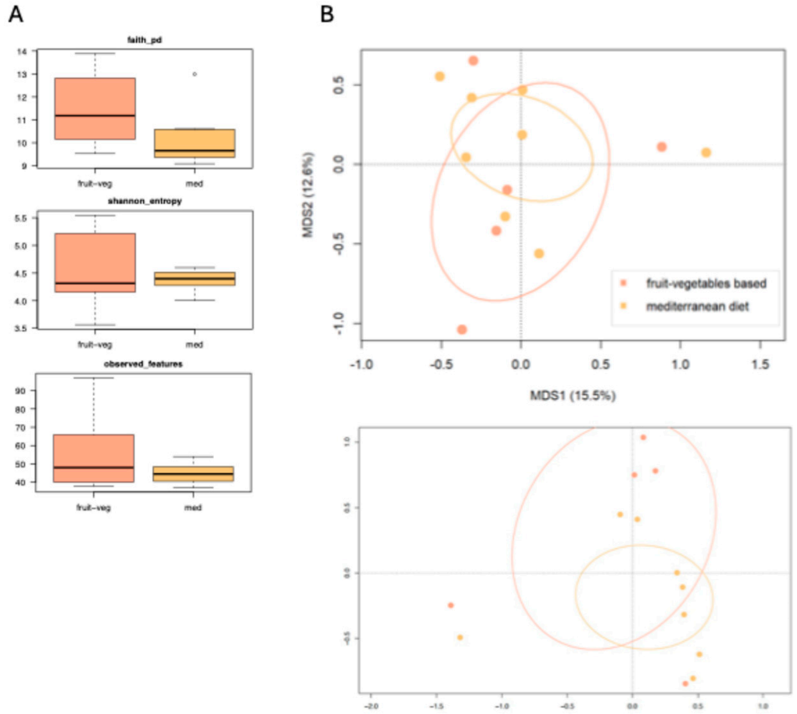

Supplementary Figure S3. Correlations between gut microbiota, dietary patterns and DICA classification in SUDD. A, Boxplots showing the distribution of alpha diversity, as estimated by Faith's phylogenetic diversity, the number of observed features and Shannon entropy, in the gut microbiota of SUDD patients scored as DICA2 who followed either a Mediterranean diet (med) or a predominantly plant-based diet (fruit-veg). No significant differences were found (Wilcoxon test, p>0.05). B, Principal Coordinates Analysis (PCoA) based on weighted UniFrac (top) and unweighted UniFrac (bottom) distances between the study groups. Ellipses include 95% confidence area based on the standard error of the weighted average of sample coordinates. No separation was found (Adonis test, p≥0.46).

Supplementary Table S1. Demographic, anthropometric and clinical characteristics of SUDD and AD patients.

	AD	SUDD	P
**Patients**	19	47	
**Male gender, n (%)**	9 (47.4%)	28 (59.6%)	0,419a
**Smoking, n (%)**	2 (10.5%)	7 (14.9%)	1a
**Median (IQR) age, years**	63.00 [54.00-71.00]	65.00 [55.00-73.50]	0,64b
**BMI, median (IQR)**	25.90 [23.96-27.00]	26.00 [24.76-30.00]	0,201b
**Diagnostic tools , n (%)**			
Colonoscopy	15 (78.9%)	38 (80.9%)	1a
Computed tomography	1 (5.3%)	6 (12.8%)	0,663a
Ecography	3 (15.8%)	3 (6.4%)	0,344a
**Symptom duration, median (IQR), months**	3.00 [0.00-4.00]	3.00 [2.00-4.50]	0,651b
**Previous appendectomy , n (%)**	6 (31.6%)	7 (14.9%)	0,172a
**Abdominal pain, median (IQR) VAS score**	0.00	5.00 [3.00-7.00]	0b
**Bristol stool form scale, median (IQR)**	4.00 [3.00-4.50]	4.00 [3.00-5.00]	0,583b

IQR: interquartile range; VAS: visual analogue scale; aFisher's exact test; bMann-Whitney test.

Supplementary Table S2. Demographic, anthropometric and clinical characteristics of SUDD patients stratified by dietary habit.

	Fruit-vegetables based diet	Mediterranean diet	Omnivorous diet	P
**SUDD patients**	8	33	6	1a
**Male gender, n (%)**	4 (50.0%)	18 (54.5%)	6 (100.0%)	0,094a
**Smoking, n (%)**	1 (12.5%)	6 (18.2%)	0 (0.0%)	0,505a
**Median (IQR) age, years**	68.00 [60.50-74.50]	65.00 [58.00-70.00]	53.00 [50.50-54.75]	0,041b
**BMI, median (IQR)**	28.50 [26.55-30.25]	26.00 [24.00-28.00]	27.85 [26.43-29.50]	0,153b
**Diagnostic tools , n (%)**				
Colonoscopy	7 (87.5%)	26 (78.8%)	4 (80.0%)	0,856a
Computed tomography	1 (12.5%)	3 (9.1%)	1 (20.0%)	0,756a
Ecography	0 (0.0%)	4 (12.1%)	0 (0.0%)	0,422a
**Symptom duration, median (IQR), months**	1.00 [1.00-2.50]	3.00 [3.00-5.00]	2.00 [2.00-5.00]	0,019b
**Previous appendectomy , n (%)**	2 (25.0%)	7 (21.2%)	0 (0.0%)	0,43a
**Abdominal pain, median (IQR) VAS score**	6.00 [4.50-6.25]	6.00 [4.00-7.00]	3.50 [3.00-4.00]	0,125b
**Bristol stool form scale, median (IQR)**	4.50 [3.00–6.00]	4.50 [3.00–6.00]	4.00 [3.00–5.00]	0.652b

IQR: interquartile range; VAS: visual analogue scale; aFisher's exact test; bKruskal–Wallis test.

Supplementary Table S3. Demographic, anthropometric and clinical characteristics of AD patients stratified by dietary habit.

	Fruit-vegetables based	Mediterranean	P
**AD patients**	7	12	
**Male gender, n (%)**	4 (57.1%)	5 (41.7%)	0,861a
**Smoking, n (%)**	2 (28.6%)	0 (0.0%)	0,237a
**Median (IQR) age, years**	72.00 [67.00–74.50]	56.00 [51.50–64.50]	0,028b
**BMI, median (IQR)**	26.00 [25.45–26.94]	25.00 [23.70–27.25]	0,582b
**Diagnostic tools , n (%)**			
**Colonoscopy**	6 (85.7%)	9 (75.0%)	1a
**Computed tomography**	0 (0.0%)	1 (8.3%)	1a
**Ecography**	1 (14.3%)	2 (16.7%)	1a
**Symptom duration, median (IQR), months**	2.50 [1.25–3.75]	3.00 [1.00–4.00]	1b
**Previous appendectomy , n (%)**	3 (42.9%)	4 (33.3%)	0,767a
**Bristol stool form scale, median (IQR)**	3.00 [3.00–5.50]	4.00 [3.75–4.00]	0,93b

IQR: interquartile range; VAS: visual analogue scale; aFisher's exact test; bKruskal–Wallis test.
